# Claudia Schlegel, editor: OSCE – Kompetenzorientiert Prüfen in der Pflegeausbildung

**DOI:** 10.3205/zma001679

**Published:** 2024-06-17

**Authors:** Elvira Pippel

**Affiliations:** 1 HejSim GmbH, Berlin, Germany

## Bibliographical details

Claudia Schlegel, editor

**OSCE – Kompetenzorientiert Prüfen in der Pflegeausbildung**

Publisher: Springer

Year of publication: 2023, 190 pages, price: € 49,99

ISBN: 978-3-662-67059-0

ISBN (eBook): 978-3-662-6706-6

## Review

OSCE exams have internationally been a staple in health profession curricula for many years. In Germany they are already established, particularly in medical education. In recent years, OSCE exams have also started to become more widespread in nursing education in Germany. Many nursing schools and universities are adopting competency-based assessment formats, but there is a lack of accompanying literature that systematically summarizes the diverse international findings, particularly addressing the specific challenges in nursing.

Now Schlegel has published the 2^nd^ ediction of a manual, which primarily serves as a guide for practical application of OSCE exams. The author explicitly states that the 190-page book is “not a scholarly treatise on OSCE exams” but aimed at all professionals involved in nursing education. In accordance with the manual's structure, it is possible to selectively read individual subsections without losing orientation. The index can be especially helpful in searching for individual OSCE stations.

The handbook is divided into two main parts: The first five sections provide an introduction to the conception of an OSCE exam, covering approximately 35 pages. This includes the advantages of OSCE exams, the development of OSCE stations, checklists and global assessment criteria, the script for standardized patients, and assessing competence levels. The OSCE stations described in the book are based on the concept of competency development according to Rauner. The first five sections provide a solid overview, particularly suitable for beginners in the subject.

The main part of the handbook, sections 6-14, focuses on the practical implementation of the OSCE exam. It presents individual OSCE stations for the first to the third year of nursing education. The topics are consistent across all three years:

1. generalist education,
2. children, adolescents, families, and women,
3. psychiatry.

First, eight steps for developing OSCE stations are presented. For each OSCE station the authors include the topic, learning objectives, task description, situation details, setting, involvement of standardized patients, a checklist, and brief practical tips. In the sections on children, adolescents, families, and women, the process for developing stations is outlined for each year of education. In the sections on psychiatry specific OSCE stations, the framing of OSCE situations corresponds to the educational level and the corresponding competency requirements in all years of education.

The most extensive part of the book covers the individual OSCE stations and their practical implementation. The diversity of case examples is well executed.

The book, referred to as a practical manual by the authors, aims to be used in the planning, development, and design of competency-based assessments. It aims to motivate the planning and implementation of OSCE exams. The book meets this aim, especially in the chapter on checklists and global assessment criteria, which is well-prepared. For beginners who are delving into the topic and need an initial overview to design stations themselves, this manual is very suitable. For advanced users, more detailed depth is needed. The book provides a good initial orientation, but further literature research on current research findings and developments, such as hybrid OSCE, standardized patients, and scripts, is lacking. Adding a description of the OSCE exam process, including resource management tips, especially for pre- and post-exam preparation, would have been useful and desirable for readers. Unfortunately, the temporal dimension for all case examples is missing, i.e., the specific time frame in which learners must complete the described stations. This information would improve planning for beginners. Additionally, an outlook and assessment by the highly experienced authors regarding current and future developments in OSCE exams would have been a useful addition to the manual.

Evidently, this handbook targets an audience in need of practical tips in the orientation phase for OSCE exam conception. The layout is clear, the structure easily understandable. Short flowing texts, graphics, and tables alternate consistently. Small practical tips are highlighted in separate colored info boxes.

In the sections covering the three educational years and different focuses, the structure varies depending on the author. The eight steps for developing OSCE stations in the “generalized education” focus illustrate the process within the OSCE exam. The sections on developing stations in focus “F (children, adolescents, families, and women)” are redundant in the second and third educational years. The general notes on the character of psychiatry specific OSCE stations in the “psychiatry” focus demonstrate the complex requirements in this area.

### Conclusion

One strength of the handbook lies in its clear and straightforward design. Graphics, tables, and info boxes break up the text and facilitate understanding. Considering the requirements for the reliability of an OSCE exam in assessing nursing competencies, the book provides an initial framework for individuals who want to implement an OSCE exam at their institution for the first time and need a practical overview. Its strength lies in describing specific OSCE stations. However, there is room for improvement, particularly for an expansion on the theoretical background and an inclusion of more current research findings. The depth of content in the theory-based section remains superficial and allows for further research and development in this area. International standards in simulation learning are not adequately cited (International Nursing Association for Clinical Simulation and Learning (INACSL) Standards, The Association of Standardized Patient Educators (ASPE) Standards of Best Practice (SOBP)). There is also a lack of an outlook on future developments of OSCE exams in nursing education.

It would have been desirable to take a comparative look at other disciplines, particularly medicine, psychology, or midwifery science. All these disciplines are already working on implementing OSCE exams, making it worthwhile to examine the transferability to the nursing profession. This could have been complemented by a focus on quality assurance of OSCE exams in addition to the explanations in sections 1-5.

This 2^nd^ edition of the book once again demonstrates how challenging it is to adapt specific OSCE stations to one's own institution. The diversity of curricula is vast, as are the basics in teaching. Developing OSCE stations requires thorough consideration of individual needs and possibilities, especially dependent on existing resources. The issue of psychological safety for learners undergoing an OSCE exam remains entirely unexplored. Not only must examiners be prepared for the OSCE exam, but the examinees themselves must also be prepared. This involves academic preparation within teaching as well as addressing the stressful nature of the OSCE exam format as perceived by learners. All those involved in the conception and implementation of OSCE exams must be aware of how the examinees feel to ensure a physically and psychologically safe exam environment. This also involves accommodations for factors such as pregnancy, language barriers, or exam anxiety.

### Outlook

The changes in nursing education are closely linked to the increasing academization of nursing. There is also a growing number of national and international publications on OSCE exams. The diversity in the implementation of OSCE exams in the German-speaking region demand a thorough examination of new developments and research findings. Therefore, there are numerous opportunities for the book to expand and deepen its coverage, especially concerning the reliability of OSCE exams, standardization of standardized patients, the use of OSCE exams as formative and summative assessment instruments, and training of examiners. The basic framework for the conception and planning of an OSCE exam as described in the present book can serve as an initial step to approach the topic.

## Note

The English version of the review was created with the help of ChatGPT.

## Competing interests

The author declares that she has no competing interests.

